# A Review of the Study Designs and Statistical Methods Used in the Determination of Predictors of All-Cause Mortality in HIV-Infected Cohorts: 2002–2011

**DOI:** 10.1371/journal.pone.0087356

**Published:** 2014-02-03

**Authors:** Kennedy N. Otwombe, Max Petzold, Neil Martinson, Tobias Chirwa

**Affiliations:** 1 Perinatal HIV Research Unit, University of the Witwatersrand, Johannesburg, South Africa; 2 School of Public Health, University of the Witwatersrand, Johannesburg, South Africa; 3 Centre for Applied Biostatistics, Occupational and Environmental Medicine, University of Gothenburg, Gothenburg, Sweden; 4 Johns Hopkins University, Center for TB Research, Baltimore, Maryland, United States of America; Istituto Superiore di Sanità, Italy

## Abstract

**Background:**

Research in the predictors of all-cause mortality in HIV-infected people has widely been reported in literature. Making an informed decision requires understanding the methods used.

**Objectives:**

We present a review on study designs, statistical methods and their appropriateness in original articles reporting on predictors of all-cause mortality in HIV-infected people between January 2002 and December 2011. Statistical methods were compared between 2002–2006 and 2007–2011. Time-to-event analysis techniques were considered appropriate.

**Data Sources:**

Pubmed/Medline.

**Study Eligibility Criteria:**

Original English-language articles were abstracted. Letters to the editor, editorials, reviews, systematic reviews, meta-analysis, case reports and any other ineligible articles were excluded.

**Results:**

A total of 189 studies were identified (n = 91 in 2002–2006 and n = 98 in 2007–2011) out of which 130 (69%) were prospective and 56 (30%) were retrospective. One hundred and eighty-two (96%) studies described their sample using descriptive statistics while 32 (17%) made comparisons using t-tests. Kaplan-Meier methods for time-to-event analysis were commonly used in the earlier period (n = 69, 76% vs. n = 53, 54%, p = 0.002). Predictors of mortality in the two periods were commonly determined using Cox regression analysis (n = 67, 75% vs. n = 63, 64%, p = 0.12). Only 7 (4%) used advanced survival analysis methods of Cox regression analysis with frailty in which 6 (3%) were used in the later period. Thirty-two (17%) used logistic regression while 8 (4%) used other methods. There were significantly more articles from the first period using appropriate methods compared to the second (n = 80, 88% vs. n = 69, 70%, p-value = 0.003).

**Conclusion:**

Descriptive statistics and survival analysis techniques remain the most common methods of analysis in publications on predictors of all-cause mortality in HIV-infected cohorts while prospective research designs are favoured. Sophisticated techniques of time-dependent Cox regression and Cox regression with frailty are scarce. This motivates for more training in the use of advanced time-to-event methods.

## Introduction

Appropriate utilization of biostatistical methods is becoming increasingly important in biomedical research. Many journals, if not all, have a dedicated statistical committee that scrutinizes the methods used in analyzing data. In the last decade, several papers addressing study design issues and statistical analysis approaches in different clinical fields have been published underpinning the importance of robustness in methodology [1]–[8]. There is consensus that inappropriate study designs and statistical methodology lead to incorrect results, poor interpretation of study findings and wrong conclusions.

An array of study designs and appropriate statistical techniques with varying levels of complexity exists. Selecting the appropriate study design and relevant statistical analysis technique is largely dependent on the complexity of the study and its objectives. Research on statistical content of medical research shows wider usage of techniques [9], [10] beyond descriptive statistics as a result of advanced software that can handle complex analyses. Much as advanced analyses are being conducted, simple techniques of descriptive and inferential statistical analysis like student t-tests and chi-square tests remain popular in the literature [4], [6], [11].

Despite major successes in the development of interventions for prevention of mother to child treatment (PMTCT) and anti-retrovirals (ARVs), HIV still remains a major public health concern. To date, limited information is available if any, reporting on the study design and statistical techniques used in determining the predictors of all-cause mortality in HIV positive cohorts in the last decade. With a large number of clinicians and public health experts relying on published research for new developments in HIV research, it is important they understand appropriateness of study designs and statistical techniques used in determining predictors of all-cause mortality. This study reviews relevant original articles in HIV-infected cohorts with the aim of identifying study designs, statistical methods used and further assess their appropriateness. We also sought to determine whether there was an increase in the use of time-to-event analysis techniques over time and highlight the need for methodological training.

## Methods

### Search strategy and selection criteria

In this bibliometric analysis, we searched all original English-language articles indexed in Pubmed/Medline using the terms “Predictors of HIV Mortality”, “Determinants of HIV Mortality” and “Factors associated with HIV mortality”. The search covered the period between January 2002 and December 2011, a period of ten years. These were further split into two five year periods; January 2002–December 2006 and January 2007 to December 2011 in order to assess whether there was a variation in the methods used over time. Original articles on HIV-infected cohorts within the specified period were eligible for inclusion. Letters to the editor, editorials, reviews, systematic reviews, meta-analysis and case reports were excluded. Other studies comparing both HIV positive and negative participants were also excluded. We identified a total of 91 and 98 papers between the periods 2002–2006 and 2007–2011 respectively.

Each article was reviewed to determine the study design, nature of statistical methods used and their appropriateness. Time-to-event analysis methods were considered optimal or appropriate in this study. A spreadsheet containing a checklist of items of interest was prepared as a data collection tool. Findings were systematically recorded based on statistical methods previously reported [12]. We used a modified version of the classification proposed by Colditz and Emerson (Table 1) [13], [14]. Where a statistical technique was used more than once in an article, we recorded it as having occurred only once. A count of the number of statistical techniques employed in each article was determined for purposes of comparing the two periods.

**Table 1:** Classification of statistical methods as reported in journals.

Category	Brief description
***t*** **-tests**	One-sample, matched pair and two-sample *t*-tests
**Contingency tables**	Chi-square tests, Fisher's exact test, McNemar's test
**Pearson correlation**	Classical product moment-correlation
**Simple linear regression**	Least squares regression with one predictor and one response variable
**Power**	Loosely defined, includes use of the size of detectable (or useful) difference in determining sample size
**Epidemiological statistics**	Relative risk, odds ratio, log odds, measures of association, sensitivity, specificity
**Adjustment and standardization**	Pertains to incidence rates and prevalence rates
**Multiway tables**	Mantel-Haenszel procedure, log-linear models
**Non-parametric tests**	Sign test, Wilcoxon signed-rank test, Mann-Whitney test
**Non-parametric correlation**	Spearman's *rho*, Kendall's *tau*, test for trend
**Analysis of variance**	Analysis of variance, analysis of co-variance and *F*-tests
**Multiple comparisons**	Procedures for handling multiple inferences on same data sets, includes Bonferroni techniques, Scheffe's contrasts, Duncan multiple range procedures, Newmann-Keuls procedure
**Transformation**	Use of data transformation (e.g logarithms) often in regression
**Multiple regression**	Includes polynomial regression and stepwise
**Life table**	Actuarial life table, Kaplan-Meier estimate of survival
**Regression for survival**	Includes Cox regression and logistic regression
**Other survival analysis**	Breslow's Kruskal-Wallis, log-rank, Cox model for comparing survival
**Cost benefit analysis**	The process of combining estimates of cost and health outcomes to compare policy alternatives
**Sensitivity analysis**	Examines sensitivity of outcome to modest changes in parameters of model, or in other assumptions
**Other**	Anything not fitting above headings, includes cluster analysis, discriminant analysis and some mathematical modeling

The statistical methods used in the research articles were classified as either parametric or non-parametric. A further classification was made describing the statistical methods used as either basic or advanced. Methods classified as basic included Student t-test, Chi-Square and Fishers Exacts test, Mann-Whitney, Kruskall-Wallis, Wilcoxon, simple one-way ANOVA and correlation statistics. Modelling approaches such as logistic Regression, Conditional Logistic Regression, Poisson Regression, Cox regression, time-varying Cox-regression and Cox regression with frailty and epidemiologic statistics were classified as advanced (Table 1).

The logistic regression is used to analyse the relationship between a binary dependent variable and independent predictor through estimation of the probability of an event occurring. It makes no assumption about normality, linearity and homogeneity of variance. But used with time-to-event outcomes, it fails to account for follow-up time. For this reason, articles reporting use of logistic regression on such outcomes were classified as sub-optimal [15]. Cox regression analysis is a survival analysis technique in time-to-event data that incorporates follow-up time and fixed covariates [16]. Censoring is done when events occur. The method assumes risk of an event is homogeneous. Extensions of the Cox regression exist which include time dependent Cox regression and Cox regression analysis with frailty [17], [18]. Time dependent Cox regression analysis accounts for the inherent correlation that may exist when covariates change over time. Cox regression analysis with frailty, if used in some of the reviewed articles, tries to account for unobserved heterogeneity.

The data collected in this study were compared between two periods. Frequency analysis was used to determine the number of studies reporting use of specified statistical techniques. The number of optimal or sub-optimal methods used in the determination of predictors of mortality was determined using frequencies. The comparison between numbers of methods reported between the two periods was compared using the chi-square test where appropriate. All the Statistical analysis was performed using SAS 9.3 software and p-values ≤0.05 were considered a significant difference.

## Results

**Figure 1:**
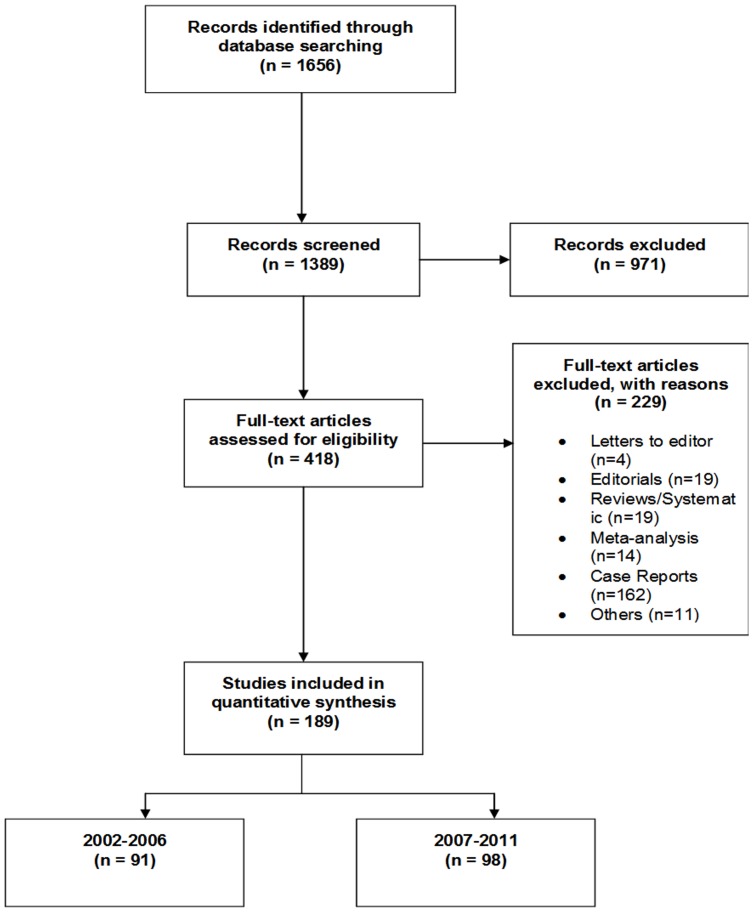
Flow chart showing the selection process of articles and the number in each period.

The total number of studies reporting on predictors of HIV mortality that met our criteria in the era January 2002 and December 2011 was 189 (n = 91 in 2002–2006 and n = 98 in 2007–2011). Figure 1 is a flow chart displaying the selection criteria that was followed in arriving at the final number of articles. All the identified articles used at least one (basic or advanced) statistical test. Journal of Acquired Immune Infection (JAIDS) (n = 34, 18%) and AIDS (n = 24, 13%) published more articles on HIV mortality. JAIDS and AIDS published 19/34 (56%) and 14/24 (58%) of these articles in the era 2002–2006. Majority of the articles used a prospective study design and the number was similar in both periods (n = 67, 74% vs. n = 62, 63%; p = 0.66). Sample sizes varied from under 200 to greater than 1,000 participants. Table 2 presents the study design and sample size distribution of the included articles between the two periods. There were no significant differences in the study designs and sample sizes used between the two periods.

**Table 2:** Types of study designs and sample sizes.

	2002–2006		2007–2011		
Research Design	No.	(%)	No.	(%)	p-value
**Prospective**	67	74	62	63	0.66
**Retrospective**	23	25	33	34	0.14
**Case control**	1	1	3	3	-
Prospective	1	-	0	-	-
Retrospective	0	-	3	-	-
**Total**	91		98		
**Sample sizes**					
up to 200	22	24	15	15	0.5
201 to 500	21	23	25	26	
501 to 1,000	16	18	20	20	
>1,000	32	35	38	39	

The number of studies reporting descriptive statistics for the two periods was similar. Table 3 presents the distribution of commonly reported statistical methods. The number of articles reporting use of t-tests, contingency table analysis, correlation and epidemiological statistics was similar. Few studies in both periods reported using one-way analysis of variance technique (ANOVA). The number of modeling approaches such as logistic, conditional logistic, generalized estimating equations and Poisson regression was significantly higher in the later period compared to the earlier (n = 31, 32% vs. n = 13, 14% p = 0.005).

**Table 3:** Summary of statistical methods.

	2002–2006		2007–2011		
Statistical methods	No. of publications (N = 91)	(%)	No. of publications (N = 98)	(%)	p-value
Descriptive statistics	88	97	94	96	0.78
Inferential methods					
t-test	13	14	19	19	0.35
Contingency table analysis					
Basic (chi-square/Fisher exact)	25	27	37	38	0.13
Correlation	9	10	1	1	-
Epidemiologic statistics	39	43	45	46	0.67
Analysis of variance	3	3	1	1	-
Regression					
#Multiple regression	13	14	31	32	0.005
Survival analysis					
Kaplan-Meier	69	76	53	54	0.002
Cox-Regression	68	75	63	64	0.12
Time dependent Cox regression	21	23	11	11	0.03
Cox-Regression with frailty	1	1	6	6	-
Non-parametric methods	86	95	91	93	0.64
No. of different inferential methods					
2 or 3 methods	10	11	11	11	0.96
4 or 5 methods	44	48	43	44	0.54
More than 5 methods	37	41	44	45	0.56
¶Basic analysis	36	40	45	46	0.38
*Advanced analysis	90	99	97	99	0.96

# Refers to logistic, conditional logistic, generalized estimating equations and Poisson regression methods.

¶ Refers to Student t-test, Chi-Square and Fishers Exact test, Mann-Whitney, Kruskall-Wallis, Wilcoxon, simple ANOVA and correlation.

* Refers to Logistic Regression, Conditional Logistic Regression, Poisson Regression and Survival Analysis and Epidemiologic statistics.

A total of 122 (65%) articles reported using the Kaplan-Meier methods and it was commonly used in the first period compared to the second (p = 0.002). Use of the Cox proportional hazards regression modeling was reported by 131 (69%) articles and the number was similar between the two eras (p = 0.12). The number reporting use of time dependent Cox regression was higher in the first period (n = 21, 23% vs. n = 11, 11%; p = 0.03). Overall Cox regression with frailty was scarcely used (n = 7, 4%) in which 6 (3%) articles were in the later period.

There were 22 (12%), 96 (51%) and 71 (38%) articles reporting use of 2 to 3, 4 to 5 and more than 5 statistical methods respectively. There were no significant differences in the number of methods used between the two eras. Similarly there were no significant differences between the two eras in the number of articles reporting use of basic or advanced statistical analysis methods.

A total of 149 (79%) of the articles used appropriate methods while 40 (21%) used sub-optimal methods to determine the predictors of mortality in HIV-infected participants. Of the articles using appropriate methods, 116 (78%) were prospective and 33 (22%) retrospective. There were significantly more articles from the first period using appropriate methods compared to the second (n = 80, 88% vs. n = 69, 70%, p-value = 0.003). Table 4 presents findings on the appropriateness of the statistical methods used. A significantly higher number of articles in the first period could have used Cox regression analysis with frailty as the appropriate method, since they had clustered data (n = 82, 92% vs. n = 65, 68%; p<0.0001) while overall they were 147 (78%).

**Table 4:** Appropriateness of statistical methods for predictors of mortality.

	2002–2006		2007–2011	
	Prospective (N = 68)	Retrospective (N = 23)	Prospective (N = 62)	Retrospective (N = 36)
Statistical methods	No. (%)	No. (%)	No. (%)	No. (%)
**Appropriate statistical analysis**				
Cox regression	53 (78)	15 (65)	48 (77)	15 (42)
Time dependent Cox regression	16 (24)	5 (22)	10 (16)	1 (3)
Cox regression analysis with frailty	1 (1)	0 (0)	3 (5)	3 (8)
Poisson regression	3 (4)	0 (0)	4 (6)	0 (0)
**Sub-Optimal statistical analysis**				
Logistic regression	4 (6)	4 (17)	7 (11)	17 (47)
Unclear	1 (1)	2 (9)	2 (3)	3 (8)

**Note:** Totals in this table do not add up to the number of articles because some articles used more than one method in their analysis.

## Discussion

This paper aimed at reviewing articles on predictors of all-cause mortality in HIV-infected people to investigate the appropriateness of statistical methods used and nature of study designs. We reviewed a total of 189 articles. Like in any other study, there were several limitations. The literature review of the articles included in this study was searched in Pubmed/Medline ostensibly because this was not a systematic review requiring a measure of effect. Any relevant articles indexed elsewhere or in a language other than English were not considered.

Our findings concur with others reporting on study designs, statistical methods used and their appropriateness. Prospective study designs remain the most common type of design used in studies of predictors of HIV mortality in the last decade. Retrospective study designs formed about one third of all articles included in this study. It may be that retrospective study designs are used as a cost-effective way of saving on huge expenses required for running prospective studies as a way for stimulating academic research. However there was no significant difference in the type of study designs used between the two periods.

Basic statistical analysis procedures like t-tests, Chi-Square and Fishers Exact tests, Mann-Whitney, Kruskall-Wallis and Wilcoxon are commonly used. There was no difference in the number of articles reporting use of t-tests between the two periods. This is similar to previously reported studies that have shown the popularity of t-tests [4], [19].

All the studies used at least two statistical tests. We contend that our inclusion criteria and the nature of studies included in this review all required using a type of statistical analysis to address the research question. Our findings concur with those reported earlier showing majority of articles apply more than one statistical test [5], [7], [20]. But this is contrary to the findings of a review on study designs and statistical methods in Chinese journals that found a low proportion of studies reporting use of multiple statistical tests [21].

Survival analysis approaches remain popular in the studies looking at predictors of mortality in HIV-infected people, especially the Cox proportional hazards regression modeling. Though fewer studies used extensions of the Cox proportional hazards regression, our findings show that there is an interest in using advanced approaches like the time-dependent Cox proportional hazards or Cox proportional hazards regression with frailty in modeling survival data in HIV-infected cohorts. We found a higher proportion of the studies could have used Cox regression analysis with frailty, an appropriate technique. While the methods used were not wrong, they could have gained more information by using Cox regression analysis with frailty. Previously reported work on statistical methods in medical research show that while use of sophisticated methods is increasing, inappropriate techniques still remain a challenge [1], [6], [7], [22]. It may be that recent techniques are advanced and require rigour to implement. Furthermore the techniques may not necessarily be easily implemented in standard statistical software [23]. As a result, researchers use techniques that are fairly straight-forward and implementable in standard statistical software.

Our findings show that not all the studies in our sample used optimal statistical tests in the determination of the predictors of mortality. Survival analysis techniques produce better estimates that are more informative when analysed using optimal methods. Furthermore, in clinical research where objectives require a multivariable analysis approach, it is prudent to adjust for confounding appropriately by using optimal statistical methods [24]. Cox regression analysis and its extensions provide a better picture compared to logistic regression when using survival data. Unlike previously reported research [21], [25], the proportion of studies using sub-optimal statistical tests was lower in our sample. These findings are contrary to those reported in other clinical fields where there was a high proportion of articles using sub-optimal methods [3], [11], [19], [26], [27].

Descriptive statistics and survival analysis techniques remain the most common methods of analysis in publications on predictors of all-cause mortality in HIV-infected cohorts while prospective research designs are favoured. These results suggest the importance of understanding advanced survival analysis methods in interpreting research findings in this set-up. However complex and appropriate methods like Cox regression analysis with frailty remain scarcely utilised. Our findings are in agreement with others who also reported a high use of descriptive statistics [4], [6]. The more sophisticated techniques of time dependent Cox regression and Cox regression with frailty are scarcely used. This motivates for more training in the use of advanced time-to-event methods.

## Supporting Information

Appendix S1 **Prisma checklist.**
(DOC)

## References

[1] Al-Benna S, Al-Ajam Y, Way B, Steinstraesser L (2010) Descriptive and inferential statistical methods used in burns research. Burns 36: 343–346. 19541424 10.1016/j.burns.2009.04.030

[2] Harris AHS, Reeder R, Hyun JK (2009) Common statistical and research design problems in manuscripts submitted to high-impact psychiatry journals: What editors and reviewers want authors to know. Journal of Psychiatric Research 43: 1231–1234. 19435635 10.1016/j.jpsychires.2009.04.007

[3] Okeh UM (2008) Statistical problems in medical research. Africa Journal of Biotechnology 7: 4819–4826. 10.4314/eajph.v6i3.45762 20088069

[4] Reed JF III, Salen P, Bagher P (2003) Methodological and Statistical Techniques: What do residents really need to know about statistics? Journal of Medical Systems 27: 233–238. 12705455 10.1023/a:1022519227039

[5] Strasak AM, Zaman Q, Marinell G, Pfeiffer PK, Ulmer H (2007) The use of statistics in medical research: A comparison of the New England Journal of Medicine and Nature Medicine. The American Statistician 61: 47–55.

[6] Taback N, Krzyzanowska MK (2008) A survey of abstracts of high-impact clinical journals indicated most statistical methods presented are summary statistics. Journal of Clinical Epidemiology 61: 277–281. 18226751 10.1016/j.jclinepi.2007.05.003

[7] Wu S, Jin Z, Wei X, Gao Q, Lu J, et al (2011) Misuse of statistical methods in 10 leading Chinese medical journals in 1998 and 2008. The Scientific World Journal 11: 2106–2114. 22125459 10.1100/2011/139494 PMC3217588

[8] Strasak AM, Zaman Q, Pfeiffer PK, Gobel G, Ulma H (2007) Statistical errors in medical research-a review of common pitfalls. Swiss Medical Weekly 137: 44–49. 17299669 10.4414/smw.2007.11587

[9] Altman DG (1998) Statistical reviewing for medical journals. Statistics in Medicine 17: 2661–2674. 9881413 10.1002/(sici)1097-0258(19981215)17:23<2661::aid-sim33>3.0.co;2-b

[10] Goldin J, Zhu W, Sayre JW (1996) A review of the statistical analysis used in papers published in clinical radiology. Clinical Radiology 51: 47–50. 8549048 10.1016/s0009-9260(96)80219-4

[11] Rigby AS, Armstrong GK, Campbell MJ, Summerton N (2004) A survey of statistics in three UK general practice journal. BMC Medical Research Methodology 4: 1–7. 10.1186/1471-2288-4-28 PMC543580 15596014

[12] Goldin J, Zhu W, Sayre JW (1996) A review of the statistical analysis used in papers published in clinical radiology. Clinical Radiology 51: 47–50. 8549048 10.1016/s0009-9260(96)80219-4

[13] Colditz GA, Emerson JD (1985) The statistical content of published medical research: some implications for biomedical education. Medical Education 19: 248–255. 4010572 10.1111/j.1365-2923.1985.tb01315.x

[14] Emerson JD, Colditz GA (1983) Use of statistical analysis in the *New England Journal of Medicine* . New England Journal of Medicine 309: 709–713. 6888443 10.1056/NEJM198309223091206

[15] Hosmer DW, Lemeshow S (2000) Applied logistic regression: John Wiley and Sons.

[16] Cox DR (1972) Regression models and life-tables. Journal of the Royal Statistical Society Series B 34: 187–220.

[17] Lin DY (1994) Cox regression analysis of multivariate failure time data: the marginal approach. Statistics in Medicine 13: 2233–2247. 7846422 10.1002/sim.4780132105

[18] Vaupel JW, Manton KG, Stallard E (1979) The impact of heterogeneity in individual frailty on the dynamics of mortality. Demography 16: 439–454. 510638

[19] Elster AD (1994) Use of statistical analysis in the *AJR* and *Radiology*: Frequency, Methods and Subspeciality Differences. AJR 163: 711–715. 8079874 10.2214/ajr.163.3.8079874

[20] Jaykaran PY (2011) Quality of reporting statistics in two Indian pharmacology journals. Journal of Pharmacology and Pharmacotherapeutics 2: 85–90. 21772766 10.4103/0976-500X.81897 PMC3127356

[21] Wang Q, Zhang B (1998) Research design and statistical methods in Chinese medical journals. Journal of the American Medical Association 280: 283–285. 9676683 10.1001/jama.280.3.283

[22] Anthony D (1996) A review of statistical methods in the *Journal of Advanced Nursing* . Journal of Advanced Nursing 24: 1089–1094. 8933272 10.1111/j.1365-2648.1996.tb02948.x

[23] Nietert PJ, Wahlquist AE, Herbert TL (2013) Characteristics of recent biostatistical methods adopted by researchers publishing in general/internal medicine journals. Statistics in Medicine 32: 1–10. 22415768 10.1002/sim.5311 PMC3521084

[24] Vähänikkilä H, Niemine P, Miettunen J, Larmus M (2009) Use of statistical methods in dental research: comparison of four dental journals during a 10-year period. Acta Odontologica Scandinavica 67: 206–211. 19308754 10.1080/00016350902837922

[25] Jin Z, Yu D, Zhang L, Meng H, Lu J, et al (2010) A retrospective survey of research design and statistical analyses in selected Chinese medical journals in 1998 and 2008. Plos One 5: 1–4. 10.1371/journal.pone.0010822 PMC2876024 20520824

[26] Lim KJ, Yoon DY, Yun EJ, Seo YL, Baek S, et al (2012) A survey of original articles published in AJR and Radiology between 2001 and 2010. Radiology 264: 796–802. 22919040 10.1148/radiol.12111976

[27] Shuai P, Zhou X-H, Lao L, Li X (2012) Issues of design and statistical analysis in controlled clinical acupuncture trials: An analysis of English-language reports from Western Journals. Statistics in Medicine 31: 606–618. 21341295 10.1002/sim.4034 PMC3631592

